# Neonatal Ventilator Associated Pneumonia: A Quality Improvement Initiative Focusing on Antimicrobial Stewardship

**DOI:** 10.3389/fped.2018.00262

**Published:** 2018-09-24

**Authors:** Anouk Goerens, Dirk Lehnick, Michael Büttcher, Karin Daetwyler, Matteo Fontana, Petra Genet, Marco Lurà, Davide Morgillo, Sina Pilgrim, Katharina Schwendener-Scholl, Nicolas Regamey, Thomas J. Neuhaus, Martin Stocker

**Affiliations:** ^1^Department of Pediatrics, Children's Hospital Lucerne, Lucerne, Switzerland; ^2^Clinical Trial Unit, University of Lucerne, Lucerne, Switzerland; ^3^Infectious Diseases Unit, Children's Hospital Lucerne, Lucerne, Switzerland; ^4^Pediatric and Neonatal Intensive Care Unit, Children's Hospital Lucerne, Lucerne, Switzerland; ^5^Pediatric Pulmonology Unit, Children's Hospital Lucerne, Lucerne, Switzerland

**Keywords:** neonatal ventilator associated pneumonia, diagnostic criteria, quality improvement, antibiotic stewardship, infection control, risk factors

## Abstract

**Background and Aims:** Neonatal ventilator associated pneumonia (VAP) is a common nosocomial infection and a frequent reason for empirical antibiotic therapy in NICUs. Nonetheless, there is no international consensus regarding diagnostic criteria and management. In a first step, we analyzed the used diagnostic criteria, risk factors and therapeutic management of neonatal VAP by a literature review. In a second step, we aimed to compare suspected vs. confirmed neonatal VAP episodes in our unit according to different published criteria and to analyze interrater-reliability of chest x-rays. Additionally, we aimed to evaluate the development of VAP incidence and antibiotic use after implementation of multifaceted quality improvement changes regarding antimicrobial stewardship and infection control (VAP-prevention-bundle, early-extubation policy, antimicrobial stewardship rounds).

**Methods:** Neonates until 44 weeks of gestation with suspected VAP, hospitalized at our level-III NICU in Lucerne from September 2014 to December 2017 were enrolled. VAP episodes were analyzed according to 4 diagnostic frameworks. Agreement regarding chest x-ray interpretation done by 10 senior physicians was assessed. Annual incidence of suspected and confirmed neonatal VAP episodes and antibiotic days were calculated and compared for the years 2015, 2016, and 2017.

**Results:** 17 studies were identified in our literature review. Overall, CDC-guidelines or similar criteria, requesting radiographic changes as main criteria, are mostly used. Comparison of suspected vs. confirmed neonatal VAP episodes showed a great variance (20.4 vs. 4.5/1,000 ventilator-days). The interrater-reliability of x-ray interpretation was poor (intra-class correlation 0.25). Implemented changes resulted in a gradual decline in annual VAP incidence and antibiotic days from 2015 compared with 2017 (28.8 vs. 7.4 suspected episodes/1,000 ventilator-days, 5.5 vs. 0 confirmed episodes/1,000 ventilator-days and 211 vs. 34.7 antibiotic days/1,000 ventilation-days, respectively).

**Conclusion:** The incidence of suspected VAP and concomitant antibiotic use is much higher than for confirmed VAP, therefore inclusion of suspected episodes should be considered for accurate evaluation. There is a high diagnostic inconsistency and a low reliability of interpretation of chest x-rays regarding VAP. Implementation of combined antimicrobial stewardship and infection control measures may lead to an effective decrease in VAP incidence and antibiotic use.

## Background and aims

Ventilator associated pneumonia (VAP) is defined as a nosocomial lower airway infection, i.e. pneumonia, in intubated patients with onset after 48 h or more of invasive mechanical ventilation^1^ (1). VAP is usually caused by airway colonization by potential pathogens, which disseminate due to inadequate immune response of the newborn's immature innate immune system. The immaturity of the immune system is especially significant in premature and growth restricted newborns (2). Sources of airway colonization can be the patient's own flora, i.e., bacterial overgrowth in oral secretions, reflux and aspiration of gastric fluid or the patient's environment with its caretakers and equipment (2, 3).

VAP is one of the most frequently diagnosed nosocomial infections (4) and, after suspected early onset sepsis, second most reason for antibiotic intervention in NICUs (5, 6). There is no international consensus on definition of VAP regarding the neonatal population (3, 7). In most recommendations radiographic changes are considered as one of the main crireria. However, underlying lung disease may complicate the interpretation of radiographic changes in this population. Reported frequency of neonatal VAP show a large range (2.7–10.9 cases per 1,000 ventilator days) in developed countries (3). VAP incidence can be reduced by infection control measures such as VAP-prevention-bundles (8–10). Epidemiologic studies demonstrated that quality improvement initiatives, not the introduction of new therapies or research approaches, resulted in a decline of mortality of neonates in the last decade (11).

Antibiotics are among the most frequently used medications in neonatal intensive care (12). There is a high variance of antibiotic use when comparing different NICUs. This observation suggests relevant overuse and underlines the need for implementation of antibiotic stewardship (13, 14). Prompt antibiotic therapy for possible infections in this vulnerable population is crucial for good outcome (15). On the other hand, inadequate antibiotic use results in increasing occurrence of multidrug resistant bacteria (16). In addition, recently published studies underline that antibiotic treatment in early life has an impact on the individual's microbiome with potential consequences for future health (17–20). Prolonged duration of antibiotic use in preterm infants is also associated with higher mortality and morbidity such as chronic lung disease, retinopathy of prematurity, periventricular leukomalacia and necrotizing enterocolitis (16, 21). Neonatologists and pediatricians should be aware that starting antibiotics may be in some circumstances more harmful than beneficial. The mind-set of antibiotic treatment just for safety reasons is no longer justified (20).

Aim of this study was to perform a review of the current literature to present an overview of the most commonly used diagnostic criteria, risk factors and therapeutic management for neonatal VAP. Secondly to analyze all suspected neonatal VAP episodes in our unit within the study period according to various predefined diagnostic criteria^1^ (22, 23). We hypothesized that a high variance in incidence between clinically suspected and confirmed neonatal VAP would exist. Additionally, because interpretation of chest x-rays is a corner stone of VAP diagnosis, we wanted to analyze the interrater-reliability of all chest x-rays done for suspected neonatal VAP episodes, hypothesizing that interrater-reliability would be modest to low. Last, we aimed to describe and compare annual incidence of neonatal VAP and antibiotic use for neonatal VAP in our NICU during a quality improvement initiative with implementation of multifaceted changes focused both on antimicrobial stewardship and infection control. We hypothesized that implementation of these changes decreases incidence of, as well as antibiotic use for neonatal VAP.

## Methods

### Literature review

The literature review was done applying the approach of the PRISMA-statement for systematic reviews (24). PubMed was searched using the following search terms: “diagnosis + neonatal ventilator associated pneumonia,” “antibiotic therapy + neonatal ventilator associated pneumonia,” and “neonatal ventilator associated pneumonia” sorted by best match, with restriction to available full text in English or German. The first search was run in December 2017, the last search was run in March 2018. In addition, further studies were identified reviewing references in found publications. Inclusion was based on the described population (only neonatal population) and the content of the study (diagnostic criteria and/or risk factors and/or management of VAP).

### Study setting

For our study, approval of the national ethics committee was obtained (Project-ID 2017-01842). The patients' parents/guardians were informed beforehand and gave consent for the study. Recordings of suspected neonatal VAP episodes as well as the single-center quality improvement initiative were undertaken at our level-III NICU in Lucerne. The Children's Hospital Lucerne is a teaching hospital for Pediatrics and Neonatology and the unit is a referral level III NICU (perinatal center) with all pediatric specialties including neonatal surgery, but without cardiac surgery (except surgical closure of persistent ductus arteriosus). The NICU is part of the Swiss neonatal collaboration with regular quality assessment and center-to-center comparison (25).

For this study, neonatal VAP was defined as VAP occurring in neonates below 44 weeks of corrected gestational age. Therefore, the study population consists of neonates with a corrected gestational age between 23 0/7 and 43 6/7 weeks hospitalized during the study period of September 1st, 2014 to December 31st, 2017. September 1st, 2014 data collection was started with implementation of a prospective surveillance program assessing VAP, central line associated blood stream infections (CLABSI) and use of antibiotics on the NICU. Every morning between 6 and 8 a.m. the on-site physician recorded patients with suspected VAP, CLABSI and patients on antibiotic treatment. The recordings were verified and entered into the NICU's surveillance database by the NICU's data manager.

### Definitions of suspected and confirmed neonatal VAP

Suspected VAP was defined according to the following criteria: ventilation for more than 48 h and new start or change of antibiotic therapy due to worsening of ventilation conditions and/or clinical deterioration and/or radiological changes compatible with pneumonia and/or changes of tracheal secretions and/or abnormal laboratory parameters (CRP > 20 mg/l, leukocytosis/-penia, I:T ratio > 0.2).

To compare the variance between clinically suspected (all episodes in our study population) and confirmed neonatal VAP, four different frameworks for diagnosis of VAP were applied on all suspected VAP episodes of our study population retrospectively. Patients fulfilling the diagnostic criteria of at least one of the frameworks were defined as confirmed VAP. Suspected episodes not fulfilling any criteria were addressed as non-confirmed VAP. The frameworks we used for further diagnosis were: 1. Center for Diseases Control and Prevention (CDC): Criteria for defining nosocomial pneumonia for infants ≤ 1 year old (22); 2. European Centre for Disease Prevention and Control (ECDC)^1^; 3. Diagnostic criteria for laboratory confirmed VAP according to a surveillance study with definition for infection specifically adapted for neonates from a Dutch NICU (23); 4. Diagnostic criteria for clinical VAP according to a surveillance study with definition for infection specifically adapted for neonates from a Dutch NICU (23). Table 1 shows a listing of all used definitions.

**Table 1:** Used definitions for diagnosing neonatal VAP.

Suspected neonatal VAP (NICU Lucerne) (Study population)	Neonates below 44 weeks of corrected gestational age Ventilation for more than 48 hours AND new start or change of antibiotic therapy due to: - worsening of ventilation conditions (increased oxygen requirements, worsening pCO_2_, increased ventilator demand) - AND/OR clinical deterioration (T > 38.0°C or < 36.5°C, P > 170/min or < 100/min, apnea >20%) - AND/OR radiological changes compatible with pneumonia - AND/OR changes of tracheal secretions - AND/OR abnormal laboratory parameters (CRP > 20mg/l, leukocytosis/-penia, I:T ratio > 0.2)
	
CDC Criteria for Infants < 1 year old (Group 1)	Patients without underlying diseases have ≥1 chest x-ray; Patients with underlying diseases have ≥2 chest x-rays with one of the following (new and persistent OR progressive and persistent): - Infiltrate - Consolidation - Cavitation - Pneumatoceles AND: - Worsening gas exchange (e.g., O_2_ desaturations, increased oxygen requirements, or increased ventilator demand) AND three of the following: - Temperature instability - Leukopenia (≤ 4000 WBC/mm^3^) or leukocytosis (>15,000 WBC/mm^3^) and left shift (>10% band forms) - New onset of purulent sputum or change in character of sputum or increased respiratory secretions or increased suctioning requirements - Apnea, tachypnea, nasal flaring with retraction of chest wall or grunting - Wheezing, rales or rhonchi - Cough - Bradycardia (<100 beats/min) or tachycardia (>170 beats/min)
	
European Centre for Disease Prevention and Control (ECDC) (Group 2)	Invasive respiratory device present (even intermittently) in the 48 preceding the onset of infection AND: - respiratory compromise AND: - new infiltrate, consolidation or pleural effusion on chest x-ray AND at least four of: - temperature >38°C or < 36.5°C or temperature instability - tachycardia or bradycardia - tachypnoea or apnoea - dyspnoea - increased respiratory secretions - new onset of purulent sputum - isolation of a pathogen from respiratory secretions - C-reactive protein > 2.0 mg/dL - I/T ratio > 0.2
	
Diagnostic criteria for laboratory confirmed VAP according to a surveillance study with definition for infection specifically adapted for neonates from a Dutch NICU (Group 3)	One of the following: - purulent sputum - changes in sputum characteristics - deterioration of ventilation conditions AND new emergence or progression of one of the following: - Infiltration - Consolidation - Pleural adhesion - Pleural effusion AND: - Isolation of a pathogenic microorganism or detection of a bacterial/viral antigen in the tracheal aspirate, bronchial secretion or sputum
	
Diagnostic criteria for clinical VAP according to a surveillance study with definition for infection specifically adapted for neonates from a Dutch NICU (Group 4)	One of the following: - purulent sputum - changes in sputum characteristics - deterioration of ventilation conditions AND new emergence or progression of one of the following: - Infiltration - Consolidation - Pleural adhesion - Pleural effusion AND: - No isolation of a pathogenic microorganism or detection of a bacterial/viral antigen in the tracheal aspirate, bronchial secretion or sputum - Administration of relevant antimicrobial therapy for at least seven days
	

### Interrater-reliability of chest-x-ray interpretation

Chest x-rays for suspected VAP episodes were ordered according to the physician in charge. The primary evaluation was done by the radiologist in charge and used for evaluation of confirmed neonatal VAP. For analysis of interrater-reliability, all chest x-rays ordered for suspected neonatal VAP were reviewed separately by all board approved neonatologists, pediatric pulmonologists and pediatric infectious disease specialists of the children's hospital of Lucerne (total of 10 senior physicians: 7 board approved neonatologists, 2 board approved pediatric pulmonologists and 1 board approved specialist for pediatric infectious diseases). They all reported if radiographic changes caused by neonatal VAP were present with a 4-point Likert scale (yes, possibly yes, possibly no, no). All x-rays were anonymized and all raters were blinded for the written interpretation by the radiologist.

### Quality improvement initiative

Within our quality improvement initiative we analyzed prospectively all episodes of suspected neonatal VAP and antibiotic use during and after implementation of multifaceted changes regarding infection control and antimicrobial stewardship in our NICU. The prospective surveillance program started in September 2014. The staff of the NICU was aware of the quality improvement initiative focused on antimicrobial stewardship, but was not informed regarding details of the ongoing analyzes for the study.

Since 2007, prescription of antibiotics in our unit is standardized by use of a web based guideline (www.idosecalc.ch) specifying drug, dose and duration of therapy (7–10 days of therapy recommended for hospital acquired pneumonia). Starting December 2015, the following multifaceted quality improvement changes were introduced over 2 years (Table 2): Firstly, a new policy for early-extubation minimizing duration of invasive ventilation was introduced in December 2015. Secondly, since December 2015, infectious disease specialists have been involved in the NICU every week for an antimicrobial stewardship round. Thirdly, a care bundle was implemented in our neonatal and pediatric ICU in December 2016, including the following measures: strict hand hygiene before and after patient contact and handling respiratory equipment, wearing gloves when in contact with secretions, ventilator circuit changes every 14 days or when visibly soiled, oral care every 2–4 h, head of bed elevation, draining ventilator condensate before repositioning of the patient, using endotracheal tube (ETT) with cuff when possible (usually not applicable for preterm neonates), choosing size of the ETT carefully to reduce numbers of reintubation.

**Table 2:** Implemented quality improvement changes in our NICU.

	**Standardized subscription of antibiotic therapies (start 2007)**	**Prospective surveillance program (start 09/2014)**	**Policy for early-extubation (start 12/2015)**	**Antibiotic stewardship rounds (start 12/2015)**	**New VAP-prevention-bundle (start 12/2016)**
2015	Yes	Yes			
2016	Yes	Yes	Yes	Yes	
2017	Yes	Yes	Yes	Yes	Yes

### Data sources

Clinical signs, oxygen requirement, ventilation requirements and further physical measurements as required according to the criteria for VAP were extracted from patient files. Antibiotic days and laboratory results were obtained from electronic patient files and reports of hospitalization. All laboratory measurements were ordered according to the request of the physician in charge and the unit's policy to use full white blood count (WBC), I:T ratio and C-reactive protein (CRP) for evaluation of suspected infection and guidance of duration of antibiotic therapy. According to the unit's policy, blood cultures and cultures of tracheal aspirates ought to be obtained before start or change of antibiotic therapy. Results were obtained from electronic patient files. Tracheal aspirates were examined microscopically with a semi-quantitative analysis of leukocytes. Purulent tracheal aspirates were defined as leucocytes ≥2 within the scale from 0 to 3.

### Statistical analyses

All data were anonymized before statistical analysis. Data collection in our NICU is generally performed for the period of a whole year (January to December), therefore annual calculations of suspected and confirmed VAP episodes and antibiotic use could only be done for the 3 complete years of the study: 2015, 2016, and 2017. The episodes in 2014 were not included in calculations as the observational period started in September.

Incidence of suspected and confirmed neonatal VAP episodes, as well as incidence of all separate groups (1–4) were calculated for the period of January 2015–December 2017 and extrapolated for 1,000 ventilator-days. Comparison of agreement between the four different diagnostic criteria was calculated using intra-class correlation coefficients (ICC). Risk factors such as gestational age, birth weight and duration of mechanical ventilation were compared between the two groups of non-confirmed and confirmed neonatal VAP. Agreement between raters for the evaluation of the chest x-rays with the 4-point Likert scale was assessed utilizing intra-class correlation coefficients (ICC). All raters evaluated all chest x-rays (fully crossed design). An ICC > 0.8 was considered as excellent, >0.6 as good agreement between raters. Annual incidence of suspected and confirmed VAP episodes and antibiotic days were calculated for 1,000 ventilator-days. To assess the existence of a possible trend regarding annual incidence of suspected VAP episodes, duration of antibiotic therapy per suspected episode and antibiotic days for suspected episodes, the nonparametric test for trend proposed by Cuzick was used (Wilcoxon-type test for trend) as the approximation works for small sample sizes (26).

## Results

### Literature review

**Figure 1:**
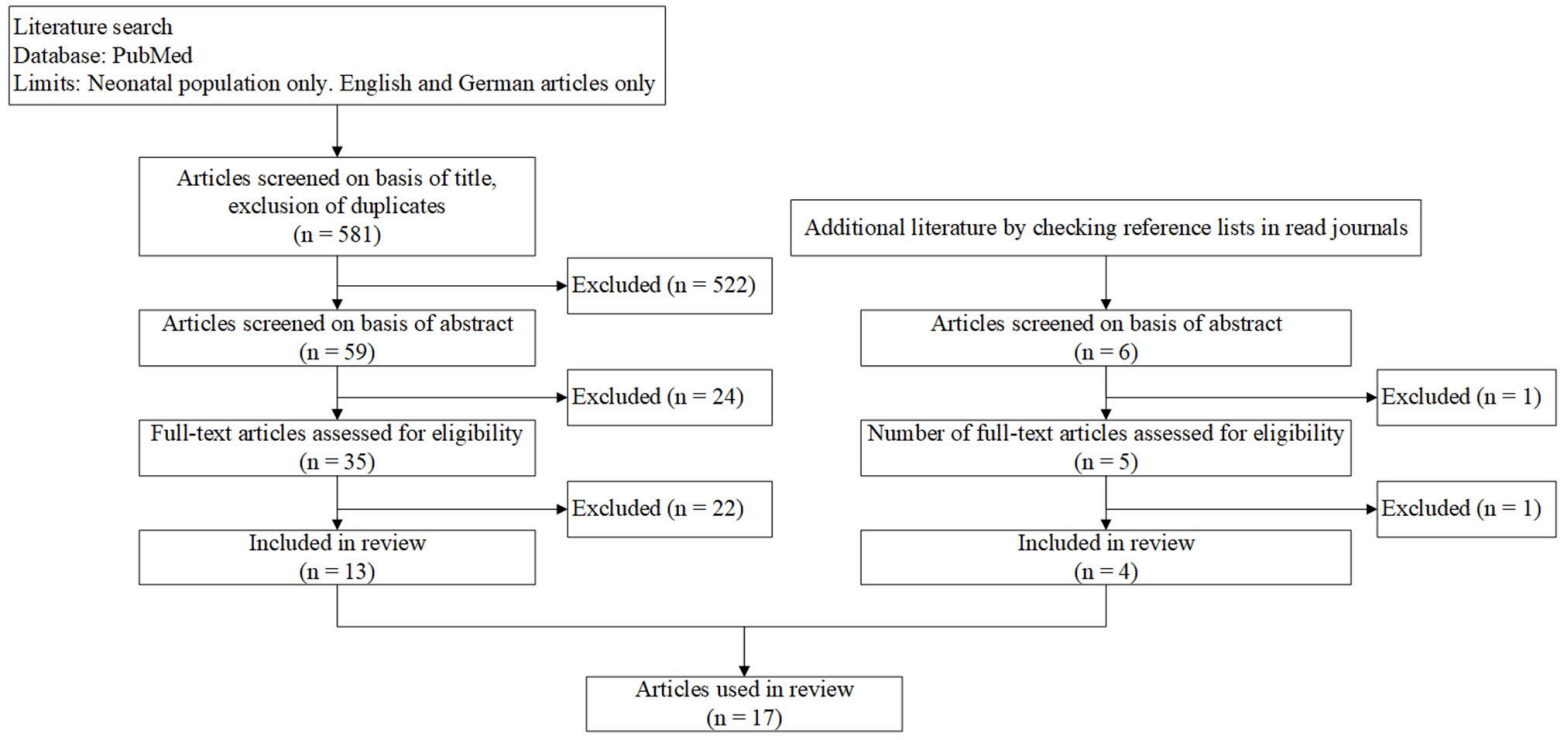
Flow-chart showing literature research.

A total of 17 studies were included in the review. The flow chart in Figure 1 shows the process of selecting studies for inclusion in the literature review. Table 3 is a listing of these recent studies, giving an overview of the used diagnostic criteria, the most important risk factors and the used therapeutic management for VAP in newborns. The CDC guidelines for infants ≤ 1 year old were the most often applied criteria followed by similar and adapted criteria. Except for the criteria used in the study by Katayama et al. (33), all request abnormal chest x-rays as part of their criteria. Longer duration of mechanical ventilation, low birth weight, low gestational age and numbers of reintubation were the prevalent risk factors for VAP. Long duration of mechanical ventilation was the most important risk factor. Even though the database was reviewed for studies regarding antibiotic therapy, only three studies (31, 33, 37) discussed the treatment of VAP.

**Table 3:** Systematic review: Overview of incidence, diagnostic criteria, risk factors and treatment.

**Source**	**Study design, (No of patients)**	**Neonatal VAP incidence**	**Diagnostic criteria**	**Risk factors**	**Treatment**
Afjeh et al. (27) Iran	Prospective cohort study (81 patients)	11.6 VAP/1,000 ventilator-days	CDC guidelines for infants ≤ 1 year old	Independent risk factors: Purulent sputum, longer duration of mechanical ventilation, antacid therapy	Not specified
Apisarnthanarak et al. (28) USA	Prospective cohort study (229 patients)	4–6.5 VAP/1,000 ventilator-days	CDC guidelines for infants ≤ 1 year old	Independent risk factors: Prior bloodstream infection, longer duration of mechanical ventilation (marginally significant)	Not specified
Azab et al. (8) Egypt	Prospective cohort study (143 patients)	73 VAP episodes	Foglia et al. (29)	Not specified	Not specified
Badr et al. (30) Egypt	Prospective observational study (56 patients)	32 VAP episodes	CPIS (clinical pulmonary infection score)	Longer duration of mechanical ventilation, low gestational age, low birth weight	Not specified
Cernada et al. (1) Spain	Prospective observational study (198 patients)	10.9 VAP/1,000 ventilator-days	CDC guidelines for infants ≤ 1 year old + positive BAL (BAL with blind protected catheter to diminish contamination)	Independent risk factor: Days of mechanical ventilation Others: Days of oxygen, times of reintubations, numbers of transfusions, previous bloodstream infection, enteral feeding, low gestational age, low birth weight, female sex	Not specified
Deng et al.(31) China	Case-control study (349 patients)	25.6 VAP/1,000 ventilator-days	At least 3 of the following: temperature instability OR new onset of purulent sputum, change in character of sputum, increased respiratory secretions, increased suctioning OR leukocytes >10x10E9 cells/l, < 3x10E9 cells/l OR two or more abnormal chest X-rays OR apnea, tachypnea, nasal flaring, grunting [Adapted Foglia et al.(29)]	Independent risk factors: Low birth weight, neonate respiratory distress syndrome, parenteral alimentation, reintubation (>3x), mechanical ventilation ≥ 7 days Others: Age < 3d, gestational age < 37 weeks, Bronchopulmonary dysplasia, previous blood stream infection, hypoxic ischemic encephalopathy, frequent drawing of blood, bronchoscopy	Cephalosporin 61.2%, Penicillin derivatives 45.5%, Aminoglycosides 13.4%, Metronidazole 20.1%, Macrolides 11.2%, Quinolones 17.8%, Vancomycin 11.6%, Sulfa derivatives 8.1%, Antifungal agents 8.9%, Antiviral agents 8.6% Duration: 5.4 ± 3.2 days
Fallahi et al. (32) Iran	Prospective cross-sectional study (66 patients)	22 VAP episodes	Modified CDC guidelines for infants ≤ 1 year old	Lower gestational age, lower birth weight, longer duration of hospital stay, prolonged ventilator need	Not specified
Katayama et al. (33) Japan	Prospective study	49 VAP episodes	Increased ventilator demand with increased amount of endotracheal aspirate + microorganisms and polymorphonuclear leukocytes in gram-stained smears of aspirates + increased CRP and/or intracellular bacteria on gram-stained smears	Not specified	Immediate gram-staining and examination of sputum aspirates by a neonatal physician: - Gram-negative bacilli: Piperacillin or Piperacillin + Amikacin - Gram-positive cocci: Vancomycin
Kawanishi et al. (34) Japan	Retrospective observational study (71 patients)	14 VAP episodes	Foglia et al. (29)	Low birth weight (esp. < 626g), times of ventilator tube changes, longer duration of mechanical ventilation	Not specified
Khattab et al. (35) Egypt	Not specified (85 patients)	47 VAP episodes (55.2%)	CDC guidelines	Prematurity, low birth weight, longer duration of mechanical ventilation	Not specified
Lee et al. (36) Taiwan	Retrospective observational study (114 patients)	7.1 VAP/1,000 ventilator-days	CDC guidelines for infants ≤ 1 year old	Longer duration of mechanical ventilation, longer parenteral nutrition, low gestational age, low birth weight	Not specified
Murila et al. (37) Australia	Retrospective study (124 patients)	74 positive ETA cultures, 58 VAP episodes	Positive culture of endotracheal secretion + overall condition + change in respiratory status (increased FiO2, increased ventilator support, new infiltrate on Chest X-ray)	Not specified	Treatment: Vancomycin + Imipenem
Petdachai (38) Thailand	Prospective observational study (170 patients)	70.3 VAP/1,000 ventilator-days	Modified CDC guidelines for infants ≤ 1 year old	Independent risk factors: Umbilical catheterization, respiratory distress syndrome, orogastric tube Others: Lower birth weight, longer duration of mechanical ventilation, longer hospital stay	Not specified
Thatrimontrichai et al. (39) Thailand	Prospective cohort study (128 patients)	10.1 VAP/1,000 ventilator-days	CDC guidelines for infants ≤ 1 year old	Independent risk factors: Birth weight < 750g, sedative medication Others: Reintubation rate, antihistamine use	Not specified
Tripathi et al. (40) India	Prospective observational study (98 patients)	37.2 VAP/1,000 ventilator-days	CDC guidelines for pediatric patients	Independent risk factors: Longer duration of mechanical ventilation, very low birth weight Others: Prematurity, numbers of reintubation, length of NICU stay	Not specified
Yuan et al. (41) China	Retrospective cohort study (259 patients)	52 VAP episodes	New and persistent radiographic evidence of focal infiltrate Plus 2 of the following: fever >38°C, leukocytes >12x10E9 cells/l, purulent sputum No hyaline membrane disease, meconium aspiration, atelectasis as possible diagnosis	Independent risk factors: Reintubation, longer duration of mechanical ventilation, treatment with opiates, endotracheal suctioning Others: transfusion, parenteral nutrition	Not specified
Van der Zwet et al. (23) The Netherlands	Retrospective surveillance study (742 patients)	5.8 - 19.7 (mean 11.8) VAP/1,000 ventilator days (depending on birth weight)	Modified CDC guidelines for infants ≤ 1 year old	Mechanical ventilation, low birthweight	Not specified

### Suspected vs. confirmed neonatal VAP

Comparison of suspected with confirmed neonatal VAP episodes showed a great variance over the whole study period: 20.4 vs. 4.5/1,000 ventilator-days. The particular incidence of confirmed neonatal VAP after applying the 4 different diagnostic criteria (groups 1–4) is shown in Table 4. Comparison of agreement between the different diagnostic criteria showed an ICC of 0.55, thus showing a moderate agreement.

**Table 4:** Incidence of suspected and confirmed neonatal VAP (in total and for groups 1 – 4).

	**Total n**	**VAP episodes/1,000 ventilator-days (2015–2017)**
**Suspected neonatal VAP episodes**	36	20.4
**Confirmed neonatal VAP episodes**		
(fulfilling diagnostic criteria for at least one out of the 4 groups)	10	4.5
Group 1 (CDC Criteria for Infants < 1 year old)	3	1.9
Group 2 (European Centre for Disease Prevention and Control (ECDC)	4	2.6
Group 3 (Diagnostic criteria for laboratory confirmed VAP according to Dutch NICU)	9	3.8
Group 4 (Diagnostic criteria for clinical VAP according to Dutch NICU)	8	3.8

**Figure 2:**
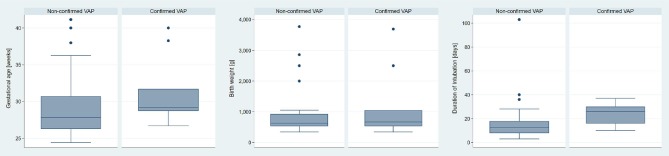
Comparison of gestational age, birth weight and duration of intubation; non-confirmed (*n* = 26) vs. confirmed (*n* = 10) neonatal VAP episodes.

The age of neonates in our study population (n = 36) ranged between 24 3/7 and 41 1/7 (median 28 4/7) gestational weeks at the time of diagnosis. They weighed between 345 and 3,770 g (median 650 g). The 26 neonates with non-confirmed VAP were between 24 3/7 and 41 1/7 (median 27 6/7) gestational weeks old and weighed between 345 and 3,770 g (median 630 g). Duration of mechanical ventilation varied between 3 to 103 days (median 12.5 days). The 10 neonates with confirmed VAP episodes were between 26 5/7 and 40 0/7 (median 29 1/7) weeks and had a birth weight between 345 and 3,690 g (median 670 g). They were intubated between 10 and 37 days (median 26 days) at time of diagnosis. Figure 2 shows a comparison of the patients' gestational age, birth weight and duration of intubation between the non-confirmed and confirmed neonatal VAP cases.

Thirty-one out of the 36 analyzed tracheal aspirates showed bacterial growth (Table 5). Thirteen of the 31 culture positive tracheal aspirates also showed a purulent sputum. Among the 10 neonates with confirmed VAP, 8 had culture positive tracheal aspirates, thereof 4 presented with purulent sputum. Table 5 shows a listing of these results and of all isolated pathogens. In 13 cases only cultures of tracheal aspirates without blood cultures were taken. Of the 23 analyzed blood cultures only one showed bacterial growth (Staphylococcus aureus). This patient also showed growth of Staphylococcus aureus in the tracheal aspirate but was not diagnosed as a confirmed VAP according to our applied criteria.

**Table 5:** Results of analyzed tracheal aspirates.

	**Purulent sputum (n)**	**Positive tracheal aspirate culture (*n*)**	**Multiple bacterial growth (*n*)**	**Pathogens**
**Suspected VAP**	13/36	31/36	8/36	
**Non-confirmed VAP**	9/26	23/26	7/26	15x gram-positive bacteria: 6x Staphylococcus epidermidis, 4x Entero-coccus faecalis, 4x Staphylococcus aureus, 1x Staphylococcus haemolyticus 12x gram-negative bacteria: 3x Escherichia coli, 2x Acinetobacter, 1x Enterobacter aerogenes, 1x Enterobacter cloacae, 1x Pseudomonas aeruginosa, 1x Klebsiella pneumoniae, 1x Klebsiella oxytoca, 1x Stenotrophomonas maltophilia, 1x Serratia marcescens Other: 2x Ureaplasma urealyticum1x Candida albicans 3x no bacterial growth
**Confirmed VAP**	4/10	8/10	1/10	4x gram-positive bacteria: 1x Staphylococcus aureus, 1x Staphylococcus haemolyticus, 1x Enterococcus faecalis, 1x Bacillus cereus 5x gram-negative bacteria: 3x Escherichia coli, 1x Pseudomonas aeruginosa, 1x Stenotrophomonas maltophilia 2x no bacterial growth

Comparison of agreement in rating all chest x-rays done for suspected neonatal VAP episodes showed an ICC of 0.25, thus showing a poor agreement between the 10 raters.

### Quality improvement initiative

**Figure 3:**
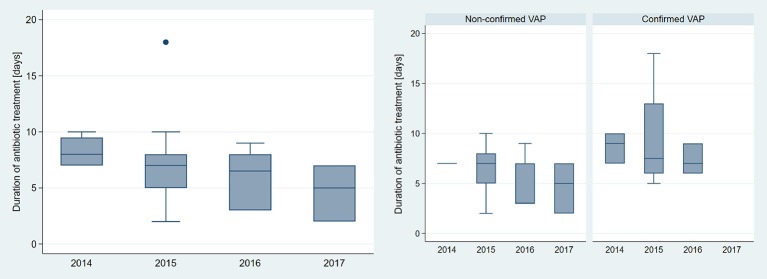
Comparison of duration of antibiotic treatment (antibiotic days/VAP episode) according to year. Left: all episodes (*n* = 36), right: non-confirmed (*n* = 26) vs. confirmed (*n* = 10) neonatal VAP.

Characteristics of the NICU's patient population for 2015 - 2017 are shown in Table 6. There were no relevant differences except a reduction in mean ventilation days per patient from 2015 to 2017. Implemented changes resulted in a gradual decline in annual neonatal VAP incidence and antibiotic days. The annual neonatal VAP incidence for 2015 vs. 2017 was 28.8 vs. 7.4 suspected episodes/1,000 ventilator-days and 5.5 vs. 0 confirmed episodes/1,000 ventilator-days (Table 7). Antibiotic days declined from 211 in 2015 to 34.7 antibiotic days/1,000 ventilator-days in 2017 for suspected episodes and from 52 in 2015 to 0 antibiotic days/1,000 ventilator-days in 2017 for confirmed episodes. Duration of antibiotic treatment of suspected VAP episodes also declined over the years with a median duration of 8 (7–10) days in 2014 vs. 5 (2–7) days in 2017 (Figure 3). Cuzick's nonparametric test for trend showed a statistically significant trend for decreasing annual incidence of suspected VAP episodes, decreasing duration of antibiotic therapy per suspected VAP episode as well as decreasing annual antibiotic days for suspected VAP episodes from 2015 to 2017 (Table 7).

**Table 6:** Annual comparison of clinical characteristics of the patient population in our NICU.

	**2015**	**2016**	**2017**
Newborns n	302	299	291
Preterm infants < 32 weeks of gestation n (%)	63 (20.9%)	76 (25.4%)	82 (28.2%)
Preterm infants < 28 weeks of gestation n (%)	22 (7.3%)	23 (7.7%)	26 (8.9%)
Mechanically ventilated newborns n (%)	97 (32.1%)	82 (27.4%)	82 (28.2%)
Ventilation days n	730	436	404
Duration (days) of mechanical ventilation (mean)	7.5	5.3	4.9
Newborns with CPAP n (%)	185 (61.3%)	199 (66.6%)	244 (83.8%)
CRIB II - Score	6.5 (±2.8)	6 (±2.8)	5.7 (±2.5)
Hospitalization days n	1,968	1,622	1,622
Mortality newborns n (%)	10 (3.3%)	6 (2%)	5 (1.7%)
Mortality preterm infants < 32 weeks of gestation, n (%)	6 (9.5%)	3 (3.9%)	3 (3.7%)
Mortality preterm infants < 28 weeks of gestation, n (%)	5 (22.7%)	3 (13%)	3 (11.5%)

**Table 7:** Incidence of suspected and confirmed neonatal VAP and antibiotic use.

	**2014 (only 09-12/14)**	**2015**	**2016**	**2017**	**Total**
**Suspected VAP episodes n**	4	21	8	3	36
Suspected VAP episodes n/1,000 ventilator-days^*^		28.8	18.3	7.4	20.4
Antibiotic days for suspected episodes n/1,000 ventilator-days^*^		211	107.8	34.7	136.9
Duration of antibiotic treatment/suspected VAP episode median in days (min – max)^**^	8 (7–10)	7 (2–18)	6.5 (3–9)	5 (2–7)	7 (2–18)
**Confirmed VAP episodes n**	3	4	3	0	10
Confirmed VAP episodes n/1,000 ventilator-days		5.5	6.9	0	4.5
Antibiotic days for confirmed episodes n/1,000 ventilator-days		52.1	50.5		38.2
Duration of antibiotic treatment/confirmed VAP episode median in days (min – max)	9 (7–10)	7.5 (5–18)	7 (6–9)		7.5 (5–18)

* *Cuzick's nonparametric test for trend p < 0.001*.

** *Cuzick's nonparametric test for trend p = 0.005*.

## Discussion

Our literature review showed that CDC guidelines for infants ≤ 1 year old or similar criteria to diagnose neonatal VAP were most often applied. These criteria are not especially adapted for the neonate or premature born population. The diagnostic criteria in 16 out of the 17 studies in our literature review requested abnormal chest x-rays. Evaluation of all chest x-rays done for suspected VAP episodes in our NICU showed a poor agreement between 10 senior physicians. This is in line with published studies describing the challenges of interpreting radiographic changes in patients with underlying lung diseases (29, 42, 43). An alternative diagnostic tool for lung pathologies is lung ultrasound (LUS). Different studies described the usefulness and accuracy of lung ultrasound for diagnosis of pneumonia in children (44, 45). Also, LUS has shown to be highly accurate for diagnosis of neonatal respiratory distress syndrome (46–48). LUS has been shown to be equivalent to chest x-rays in these studies. Hiles et al. (47) even described less intra-observer discrepancy in identification of small pneumonias. Further studies concerning sonographic diagnosis of pneumonia in the neonatal population are needed.

Additionally, unspecific clinical and laboratory findings challenge further the correct diagnosis of neonatal VAP (3, 49). Existence of purulent sputum is part of most used diagnostic criteria for VAP^1^ (22, 23). Only 4/10 of our confirmed neonatal VAP episodes showed purulent sputum. On the other hand, 31 out of all 36 neonates and 23 of the 26 non-confirmed VAP episodes showed bacterial growth in the analyzed tracheal aspirate. This might be caused by a high rate of colonization without clinical relevance. Cultures of tracheal aspirates are routinely taken if VAP is suspected although they are not part of most diagnostic criteria (37). Other authors described the frequent occurance of colonized tracheal aspirates (37, 50). A study by Ruiz et al. (51) showed no significant difference in diagnostic accuracy between noninvasive (tracheobronchial aspiration) vs. invasive (fiberoptic bronchoscopy with protected specimen brush and bronchoalveolar lavage) investigation techniques regarding VAP, thus not supporting more invasive procedures with more side effects. A study comparing treated vs. untreated episodes of culture-positive endotracheal aspirates showed that treated episodes also showed worse ventilation conditions, clinical symptoms and laboratory parameters (37), highlighting the limited usefulness of one diagnostic parameter by itself.

**Figure 4:**
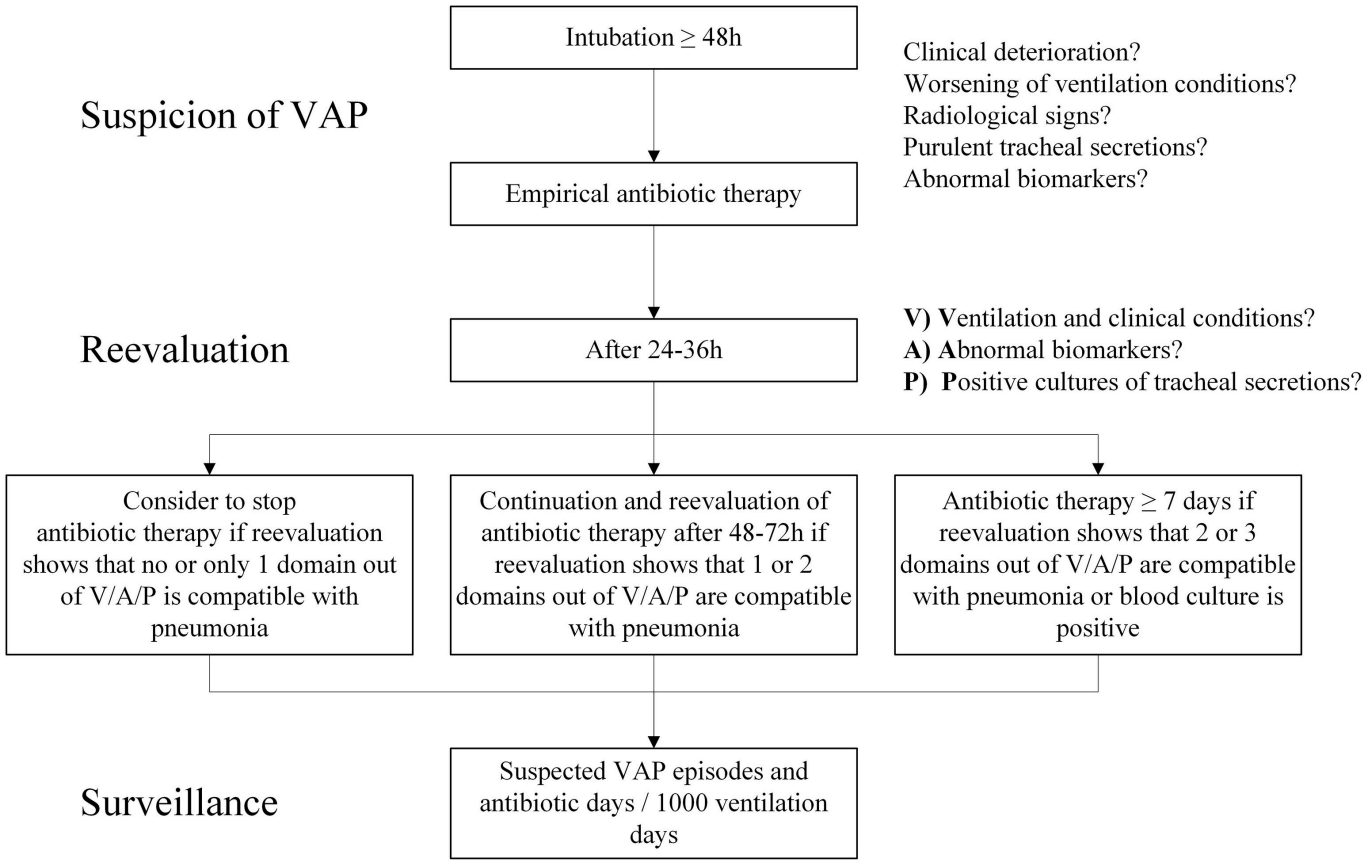
Possible algorithm to approach suspected VAP.

The moderate correlation for diagnosis of VAP according to 4 different frameworks in our study underlines the diagnostic problem. Incidence of VAP is often used as quality indicator and the discussed insecurity regarding diagnosis is a potential bias (49). Our findings, consistent with the published literature regarding the difficulty to diagnose neonatal VAP, demand an adaption of current diagnostic criteria for the neonatal population. On the other hand, due to the critical illness of neonates on the ventilator, early start of empiric antibiotic therapy for suspected VAP is mandatory. The challenge is to reevaluate empiric therapy after 24 to 36 hours combining clinical, laboratory and cultural findings. Figure 4 shows a possible algorithm to approach suspected VAP.

Comparison of incidence between suspected and confirmed neonatal VAP episodes in our NICU showed a 4.5 times higher rate for suspected vs. confirmed VAP episodes/1,000 ventilator-days (20.4 vs. 4.5). Our rate of 4.5 confirmed VAP episodes/1,000 ventilator-days over the whole study period is in line with the published incidence in the studies in our systematic review (2.7–11.8 in developed countries). Prompt antibiotic therapy for possible infections is important for optimal outcome in the neonate population (15) and newborns with suspected VAP are often treated empirically without reviewing defined criteria for VAP (6). Median duration of antibiotic treatment for suspected and confirmed VAP episodes in our cohort was 7 (2–18) and 7.5 (5–18) days, respectively. Also, Cantey et al. recently reported a wide range of antibiotic treatment duration for culture-negative neonatal pneumonia from 5 to 14 days (median 7 days) (5). Taking into account antibiotic treatment for all episodes of suspected neonatal VAP, overall antibiotic use is much higher than reported for confirmed VAP. Our results show a 3.5 times higher number in antibiotic days when including all suspected VAP episodes. We conclude that an inclusion of suspected VAP episodes should be considered for accurate assessment and quality control of VAP incidence and antibiotic use in the neonatal population.

Implementation of multifaceted changes regarding antimicrobial stewardship as well as infection control in our NICU resulted in an annual decrease of suspected and confirmed VAP episodes. Infection control measures are aimed at the prevention of nosocomial or healthcare-associated infections (52). Methods used for infection control include hand hygiene, isolation guidelines, handling and disinfection of patient care equipment, instruments and devices and use of personnel protective equipment (53). Introduction of VAP-care-bundles to prevent infections has already been done in different units showing a potential significant reduction of VAP episodes after implementation (8, 9). Furthermore, our changes resulted in an important annual decline in overall antibiotic days and treatment days per VAP episode. Antimicrobial stewardship aims to reduce inappropriate antibiotic therapy by improving selection, duration, dosage and application route of drugs (54). A systematic review by Kaki et al. (54) showed that implementation of antimicrobial stewardship interventions in different hospitals resulted in reduction of overall antibiotic use, inappropriate therapy, duration of therapy and adverse events toward antibiotics. A recently published antibiotic stewardship study by Cantey et al. (5) reported a safe reduction of antibiotic use for pneumonia in a NICU population. The implemented changes in our unit not only reduced confirmed neonatal VAP episodes, but also the numbers of suspected episodes. This resulted in a 6-fold reduction of at least partly unnecessary antibiotic days per 1,000 ventilator-days (211 vs. 34.7/1,000 ventilator-days for 2015 vs. 2017) and a statistically significant trend for decreasing duration of antibiotic therapy and antibiotic days in total over time from 2015 to 2017. Both, reduction of antibiotic days as well as declining VAP incidences resulted in this trend, with reduction of VAP episodes statistically being the more important composite. As several changes were initiated at various time points, it is not possible to distinguish the impact of a single measure. We assume that both, infection control and antibiotic stewardship measures together had an impact on the observed reduction in incidence of neonatal VAP and antibiotic use: antimicrobial stewardship measures by increasing awareness toward antibiotic therapy, and infection control measures by reducing VAP episodes and thus antibiotic therapy. A position paper published by the Association for Professionals in Infection Control and Epidemiology (APIC), the Society for Healthcare Epidemiology of America (SHEA), and the Society of Infectious Disease Pharmacists (SIDP) stated the importance of joining antimicrobial stewardship and infection control measures, as implementation of a combination of both is more effective than one measure by itself (10).

All studies in our systematic review state long duration of mechanical ventilation as a risk factor for developing neonatal VAP. Comparing patient population of non-confirmed vs. confirmed VAP episodes, confirmed episodes in our NICU were also associated with longer duration of mechanical ventilation. Studies by Hentschel et al. (55) and Geffers et al. (56) showed lower pneumonia rates in patients ventilated with CPAP-devices compared to intubated patients. These studies support the strategy toward early extubation. Implementation of our early-extubation policy in our unit resulted in a decrease of duration of invasive ventilation without increase of mortality.

There are several limitations to our study: Firstly and most important, this is not an intervention study with a control group aiming to prove the causative benefit of the implemented changes. Nevertheless, the reduction of incidence and antibiotic use for neonatal VAP is remarkable. The NICU's patient population did not change remarkably over the three years and there were no other changes over the study period applied. Therefore, we could not determine any other apparent reason for the decline in numbers. Secondly, the sample size of VAP episodes is small and therefore it was not possible to further analyze and compare more risk factors or clinical indicators. Also numbers for confirmed VAP episodes are only descriptive as no further statistical calculations were done due to the even smaller sample size. Thirdly, whereas all VAP episodes were collected prospectively, diagnostic analyzes according to the 4 frameworks were done in retrospect.

## Conclusions

Diagnosis and confirmation of neonatal VAP is difficult, resulting in a great variance between suspected and confirmed episodes. An adaption of current diagnostic criteria for the neonate population might be helpful for more consistency. Inclusion of both confirmed and suspected episodes should be considered for accurate evaluation and comparison of VAP incidence and antibiotic use in the neonatal population as quality indicators. Implementation of combined antimicrobial stewardship and infection control measures may lead to an effective decrease in both VAP incidence and antibiotic use.

## Ethics statement

The study was carried out in accordance with the recommendations of the national ethics committee (Ethikkommission Nordwest- und Zentralschweiz EKNZ: Project-ID 2017-01842) with informed consent from patients' parents/guardians. The EKNZ exempt to have a written consent due to the fully anonymized data and the quality improvement focus of the study without need of additional information than obtained from patient files.

## Author contributions

MS devised the project, details of implementation were discussed between MS and AG. Data collection and writing of the manuscript was done by AG, closely revised by MS. Statistical calculations were done by DL. The literature review was done by AG. Interpretation of chest-x-rays was done by NR, MB, ML, KD, KS-S, SP, PG, DM, MF, and MS. Critical revising of the work was done by TN, NR, MB, ML, KS-S, SP, PG, DM, MF, and KD. All authors read and approved the submitted version of this manuscript.

### Conflict of interest statement

The authors declare that the research was conducted in the absence of any commercial or financial relationships that could be construed as a potential conflict of interest.

## Footnotes

^1^*0512-TED-PPS-HAI-antimicrobial-use-protocol*. Available online at: https://ecdc.europa.eu/sites/portal/files/media/en/publications/Publications/0512-TED-PPS-HAI-antimicrobial-use-protocol.pdf (Accessed September 16, 2017).

## Abbreviations

CDC: Center for Diseases Control and Prevention
CLABSI: Central line associated blood stream infections
CPAP: Continuous positive airway pressure
CRIB: Clinical risk index for babies
CRP: C-reactive protein
ECDC: European Centre for Disease Prevention and Control
ETA: Endotracheal aspirate
ETT: Endotracheal tube
I:T ratio: Immature neutrophils to total neutrophils
ICC: Intra-class correlation coefficient
LUS: Lung ultrasound
NICU: Neonatal intensive care unit
VAP: Ventilator associated pneumonia
WBC: White blood cell count.
